# Disease in the Sewing Machine

**Published:** 1889-08

**Authors:** 


### DISEASE IN THE SEWING MACHINE.

The sewing machine has not only produced a revolution in the method of working with the needle and thread, but it has also created a special class of nervous diseases among the workers. It is not difficult to understand how the mechanical action of turning the sewing machine with the feet causes functional derangements. Nervous affections are also very common among the workers who are thus employed. The "jarring" causes in the young and weekly, extreme nervous irritation and depression, headache and restlessness. Cases have come under our notice in which health has been permanently injured by long and continued work of this description. In factories, the labor of turning the sewing machine—labor which, when badly constructed machines have to be used, is very considerable, and with all machines very irksome—should not be thrown on the workers, many of whom are mere children, since a very small amount of steam or electric power would turn a large number of the machines. The girls, by being able to concentrate their attention on the work, would direct it with more care, and the masters would lose little by doing that which would protect the health of their workwomen. We know that either steam or electric power is employed in many establishments in this city. One or the other should be in all.

Elecrric power is preferable, where obtainable, as it avoids the heat and dust, and unwholesome odors inseparable from the use of steam.

Cheap and economical motors are now made for such purposes, and the power is led into the buildings from adjacent electric light wires at small cost.

Women now do much work formerly done by men ; and nearly all the cheap clothes are the work of women's hands. Many of the workwomen suffer from the effects of overcrowding in badly ventilated workrooms. The girls become sick and faint, and scores of them apply for treatment in the hope that medicine will remedy the maladies—some slight, some grave, which the sanitary defects of the workrooms, and fatigue of turning and the vibration of the machines have caused.

